# Handicap-Recover Evolution Leads to a Chemically Versatile, Nucleophile-Permissive Protease

**DOI:** 10.1002/cbic.201500295

**Published:** 2015-07-14

**Authors:** Thomas Shafee, Pietro Gatti-Lafranconi, Ralph Minter, Florian Hollfelder

**Affiliations:** a Department of Biochemistry, University of Cambridge 80 Tennis Court Road, Cambridge, CB2 1GA (UK); b Antibody Discovery and Protein Engineering, MedImmune Granta Park, Cambridge, CB21 6GH (UK); c Present address: La Trobe Institute of Molecular Sciences, La Trobe University, Science Drive Melbourne, Victoria 3086 (Australia)

**Keywords:** directed evolution, nucleophilic catalysis, PA clan, proteases, tobacco etch virus

## Abstract

Mutation of the tobacco etch virus (TEV) protease nucleophile from cysteine to serine causes an approximately ∼10^4^-fold loss in activity. Ten rounds of directed evolution of the mutant, TEV^Ser^, overcame the detrimental effects of nucleophile exchange to recover near-wild-type activity in the mutant TEV^Ser^X. Rather than respecialising TEV to the new nucleophile, all the enzymes along the evolutionary trajectory also retained the ability to use the original cysteine nucleophile. Therefore the adaptive evolution of TEV^Ser^ is paralleled by a neutral trajectory for TEV^Cys^, in which mutations that increase serine nucleophile reactivity hardly affect the reactivity of cysteine. This apparent nucleophile permissiveness explains how nucleophile switches can occur in the phylogeny of the chymotrypsin-like protease PA superfamily. Despite the changed key component of their chemical mechanisms, the evolved variants TEV^Ser^X and TEV^Cys^X have similar activities; this could potentially facilitate escape from adaptive conflict to enable active-site evolution.

Enzymes achieve efficient catalysis through the precise orientation of a key set of active-site residues. This arrangement is dependent on chemical constraints, to the extent that some active-site geometries have convergently evolved many times.^1^ Consequently, active-site residues are the most evolutionarily conserved within enzyme families. Phylogenetic analysis of extended protein superfamilies suggests that even residues that are crucial for activity are exchanged during evolution over sufficiently long timescales.^2^ The evidence for such exchanges raises the question of how a gene coding for an inefficient enzyme can persevere in the transition from one type of active site to another. There is no evolutionary advantage for maintaining a gene coding for a catalytically impaired or inactive protein, thus creating the scenario of “adaptive conflict”.^3^ We know from studies on enzymes^4^ and enzyme models^5^ that precise positioning is easily disturbed. Minute disturbances down to the picometer scale cause substantial rate reductions.^6^ Given the delicacy of catalytic arrangements, it is unknown how the evolution of active sites avoids unfit variants.

Serine and cysteine proteases are textbook examples of enzymes employing covalent, nucleophilic catalysis (Figure 1 A)^7^ that leads to substantial rate acceleration of a difficult reaction (with a half-life of ≈500 years).^8^ The sophisticated interplay of the multiple active-site residues involved, including for example, the charge relay system of the catalytic triad,7c suggests that any deviation from such a highly efficient arrangement is likely to be penalised.^9^ However, phylogenetic analysis of proteases suggests that nucleophile exchanges do occur during evolution. The PA clan of chymotrypsin-like proteases^10^ encompasses both serine and cysteine proteases evolved from a hypothetical common ancestor.^11^ Constructing a phylogeny of this clan of proteases (Figure 1 B) shows that—within a highly conserved structure—nucleophile switches must have occurred by divergent evolution at least once: cellular proteases use a serine nucleophile, but both cysteine and serine protease families are found in viruses.^12^

**Figure 1:**
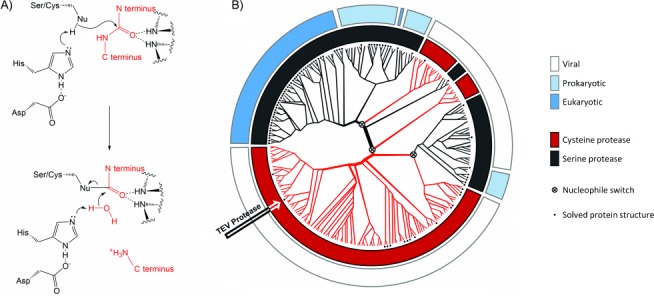
A) Simplified catalytic mechanism of cysteine and serine proteases (Nu=S, e.g., in TEV^Cys^; or Nu=O, e.g., in TEV^Ser^).^7^ In the enzyme’s catalytic triad (black) aspartate aligns and polarises histidine, which reduces the nucleophile’s p*K*_a_ and positions it for reaction. The activated nucleophile attacks the carbonyl of the peptide bond of the substrate (red), a tetrahedral intermediate is stabilised by backbone amide hydrogens (“oxyanion hole”), and the C terminus of the substrate is ejected, leaving a covalent adduct behind. This acyl–enzyme intermediate (lower panel) is hydrolysed by histidine-activated water to release the N terminus of the substrate (aided by proton transfer to histidine), and this regenerates the free enzyme. B) The phylogeny of the PA clan (MEROPS classification)^11^ of proteases with nucleophile and possible nucleophile-switching events indicated. Proteases with known structures were aligned to TEV^Cys^ by structural comparison using DALI,^13^ sequences of unknown structure were added by sequence alignment with BLASTp (see Table S1 and Figure S1 for details).

When the active-site nucleophiles of serine or cysteine protease are interconverted, the single atomic change typically leads to a >10^4^-fold reduction in *k*_cat_*/K*_M_.9a, 14 Although both thiol and hydroxy groups can act as nucleophilic catalysts, their positioning is likely to be suboptimal after mutation due to structural differences between oxygen and sulfur: oxygen’s smaller atomic radius (by ∼0.4 Å)14a and the formation of shorter bonds (decreasing *d*_C–X_ and *d*_X–H_ by ∼1.3-fold each) would be expected to disturb the precise nucleophile positioning. In addition there are reactivity differences: sulfur is softer, and its different p*K*_a_ (4–5 units lower for RSH compared to ROH) provides a larger fraction of the active form of the nucleophile at physiological pH; this explains why the reactivity of the hydroxy side chain of serine that replaces cysteine would be compromised.14d

These considerations of chemical reactivity and structure raise the question of how such nucleophile transitions have occurred in proteases, despite the enzyme inactivation typically associated with mutating a key active-site residue. Handicap-recover experiments can be used to find if any mutations can epistatically offset a known deleterious mutation or the replacement of a native cofactor.^15^ Here we use this approach to demonstrate a scenario that could satisfy the fitness requirements of protein evolution by mutating the crucial nucleophile of tobacco etch virus cysteine protease (TEV^Cys^) to serine (TEV^Ser^), recovering activity by directed evolution (DE) and measuring trade-offs in response to nucleophile switches.

The nucleophile mutation compromised the centrepiece of the catalytic mechanism and consequently had a much greater effect on activity than in previous handicap-recover experiments.^15^ A TEV^Ser^ mutant (C151S mutation) was constructed, and the substitution of the cysteine thiol nucleophile by a serine alcohol was found to reduce activity by four orders of magnitude (Figure 2 and Figure S4 in the Supporting Information). The effect of this deliberately introduced handicap was then quantified by measuring the reaction kinetics (by monitoring the hydrolysis of the C-Y FRET-pair substrate,^16^ Figure S2). Conversion of the catalytic nucleophile from sulfur to oxygen resulted in biphasic kinetics (Figures 2 A and S3, Table S2); this is consistent with the formation and breakdown of an acyl–enzyme intermediate (i.e., a fast first step followed by a slower, rate-limiting step, Figure S5). The second-order rate constants ${k{{{\rm obs1}\hfill \atop 2\hfill}}}$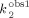 and ${k{{{\rm obs2}\hfill \atop 2\hfill}}}$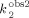 of TEV^Ser^ were found to be 80 and 20 000 times lower, respectively, than the measured second-order rate constant of TEV^Cys^ (representing *k*_cat_*/K*_M_).

**Figure 2:**
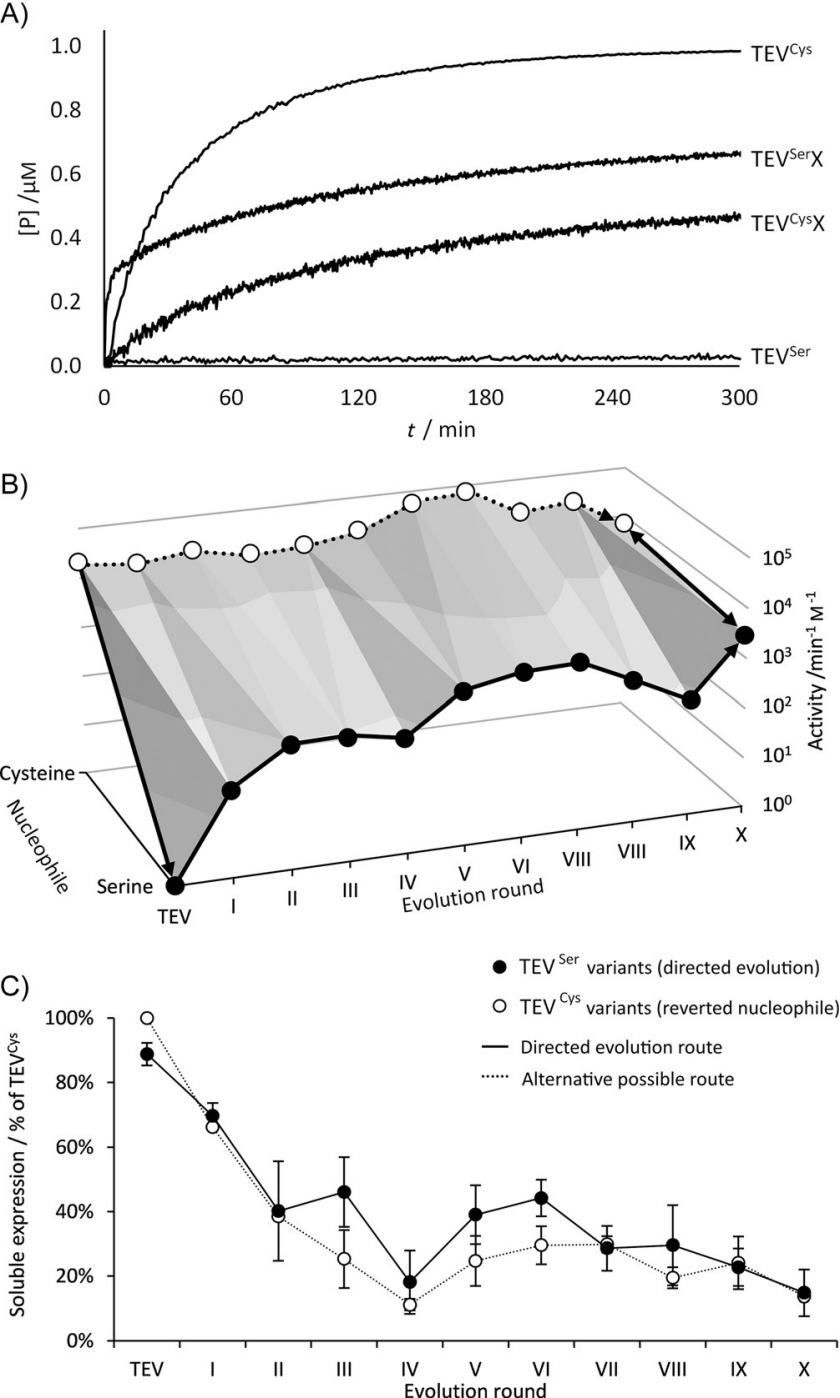
Activity recovery of the directed evolution lineage from TEV^Ser^ to TEV^Ser^X and the corresponding nucleophile revertants, in which the original Cys nucleophile was restored. A) Kinetic traces of enzyme activity for the wild type-type enzyme (TEV^Cys^), the nucleophile mutant (TEV^Ser^), the evolved enzyme from the tenth round (TEV^Ser^X) and the evolved mutant with a reverted nucleophile (TEV^Cys^X). Conditions: [E]=1 μM, [S]=1 μM, pH 8, 25 °C. B) Development of the activity of purified TEV mutants as a function of the rounds of directed evolution. Activities are plotted as the second order rate constants ${k{{{\rm obs2}\hfill \atop 2\hfill}}}$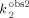 of the ten TEV^Ser^ variants and *k*_cat_*/K*_M_ of the ten TEV^Cys^ nucleophile revertants. Solid arrows indicate the DE route (starting with the deleterious TEV^Cys^→TEV^Ser^ mutation, then activity recovery in ten rounds from TEV^Ser^ to TEV^Ser^X) to arrive at the neutral twin enzymes TEV^Ser^X and TEV^Cys^X, capable of using either nucleophile. The dotted arrow indicates an alternative, nearly neutral TEV^Cys^→TEV^Cys^X route. Conditions: [E]=1–8 μM, [S]= 1 μM, pH 8, 25 °C. Standard deviations of four repeats were below 15 % (Figure S5). C) Soluble expression of evolved variants and TEV^Cys^ back-mutants. Error bars represent standard deviation of two biological repeats.

In order to investigate how the enzyme can compensate for the handicap of using a non-native nucleophile and altered reaction chemistry, ten rounds of DE for activity recovery were performed (numbered TEV^Ser^ to TEV^Ser^X). Each round of DE consisted of error-prone PCR (1.3±0.4 amino acid mutations per gene), activity screening of 350 enzyme variants by destruction of FRET in cell lysate (Figure S2), and selection of the single best variant. Any S151C revertants were discarded to force evolution to follow a forward pathway. Measurement of turnover rates in cells during screening reflects enzyme fitness as the product of both chemical reactivity and catalyst concentration (determined by biophysical properties, such as folding and solubility). The same FRET-pair substrate was used for both in vivo screening and in vitro kinetics to describe the enzyme–substrate interactions that were relevant for the selection (and avoid unique effects of the recognition of for example, small-peptide substrates with different reactivity and affinity).

During the rounds of evolution, no mutations in residues that make direct contacts with the triad (or are within a radius of 4 Å) resulted from experimental selections. Conversely, nine of the 13 point mutations accumulated 4–8 Å from the catalytic triad, in the second shell of residue interactions (Figure 3). In vitro kinetics of purified variants showed that the process of directed evolution recovered proteolytic activity by an improvement in both ${k{{{\rm obs1}\hfill \atop 2\hfill}}}$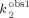 (2×10^3^-fold) and ${k{{{\rm obs2}\hfill \atop 2\hfill}}}$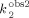 (3×10^3^-fold) and also changed the burst amplitude (Figure 2 A, Tables S2 and S3), with diminishing improvements in later rounds. The accumulation of mutations around the enzyme active site (second shell) that increased catalysis reduced soluble expression sixfold; however, in rounds V and VI, surface mutations (W130C and E194D) and a C-terminal truncation (due to a frameshift) were selected that improved both solubility and activity (Figure 2 C).

**Figure 3:**
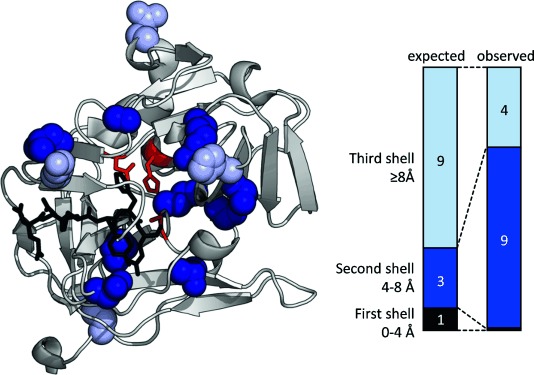
Positions of mutations accumulated during directed evolution mapped onto the structure of TEV protease (PDB ID:1LVM). The catalytic triad is shown in red, the substrate peptide is in black. There are no mutations within 4 Å of the catalytic triad (the first shell), mutations 4–8 Å from the triad (the second shell) are shown in dark blue, and mutations 8–12 Å from the triad (the third shell) are shown in light blue. Mutations in the second shell around the catalytic triad are enriched compared to what would be expected from a random distribution.

In addition to the adaptive trajectory of TEV^Ser^ (Figure 2 B, front), the identified adaptive mutations were examined in the parental background, by reverting the nucleophile to the original cysteine at each step of the evolutionary trajectory (Figure 2 B, back). The kinetics of these TEV^Cys^ variants could be fitted to a monophasic model with good correlation coefficients. Rather than respecialising the active site to use serine, as is typical of directed-evolution experiments, the 14 mutations accumulated by DE proved nearly neutral to activity with the original cysteine nucleophile (i.e., did not trade-off). Whereas the ${k{{{\rm obs1}\hfill \atop 2\hfill}}}$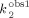 and ${k{{{\rm obs2}\hfill \atop 2\hfill}}}$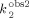 of TEV^Ser^X are 1000-fold improved over those of TEV^Ser^, the nucleophile revertants retained activity within fourfold of TEV^Cys^ (Figures 2 B and S6). The evolutionary trajectory therefore results in twin enzymes, differing only in their nucleophile (TEV^Ser^X↔TEV^Cys^X) and with only a small, 2.3-fold difference in activity upon nucleophile exchange.

By forcing TEV into a local fitness valley (TEV^Ser^) and experimentally evolving for activity recovery, we mapped out an uphill trajectory (TEV^Ser^→TEV^Ser^X) that lies parallel in sequence space to a nearly flat, neutral trajectory of mutants with constant Cys nucleophile (TEV^Cys^→TEV^Cys^X; Figure 2 B). Therefore, despite deliberately evolving TEV^Cys^ by using a fitness valley, the nucleophile-permissive TEV^Ser^X can also be accessed from TEV^Cys^ by nearly neutral mutations without any large drops in activity. The most closely related natural serine protease only retains 15 % sequence identity to TEV^Cys^. However, the mutant TEV^Ser^X shows 92.4 % identity (Table S2, Figure 3), thus suggesting that only a few mutations are necessary to accommodate a nucleophile switch. The >1000-fold improvement to generate a nucleophile generalist with only 13 mutations explains how divergent evolution of core catalytic machinery can occur within evolutionarily superfamilies such as the PA clan (Figure 1 B). It also emphasises the power of directed evolution to find solutions for the challenge of retuning chemical reactivity.^17^

The challenge of nucleophile permissiveness is conceptually similar to that of catalytic promiscuity (the ability of an enzyme to accept different substrates): how can an enzyme make and break bonds different from those it has evolved for?^18^ The evolution of promiscuous activities has been previously observed to pass through catalytic generalists, which were able to promote a new reaction, while retaining some activity on their original substrate. Generalist enzymes are proposed to perform an important role in the evolution of new functions by being particularly evolvable.^19^ Although enzyme promiscuity towards different substrates is well documented,^18^, ^20^ the ability to use different residues for nucleophilic, covalent catalysis represents an alternative kind of chemical versatility in the core catalytic machinery.^21^

Even though the lynchpin of catalysis in the protease active site—the nucleophile—was mutated, the large activity drop was readily recoverable by evolution. Quantitatively, both the handicap introduced and the extent of the recovery exceed those previously observed by approximately two orders of magnitude.^15^ Specifically, a 120-fold reduction triggered by cofactor exchange was followed by a 70-fold recovery in a study by Miller et al.15a and a 400-fold reduction caused by mutation of a conserved residue was followed by a 40-fold recovery observed by Wellner et al.,15b compared with a 20 000-fold reduction and 3000-fold recovery in this work.

What is more, the similar rates of TEV^Cys^X and TEV^Ser^X (3×10^3^ vs. 7×10^3^ min^−1^ M^−1^) represent a rare example of catalytic promiscuity at high, wild-type levels (in contrast to promiscuous, yet low-activity catalytic generalists).^22^ Paradoxically, a nucleophile mutation would be predicted to be more difficult to recover from, when compared to evolution to accommodate promiscuous binding of multiple substrates, as two different types of bonds (O−C vs. S−C) are being formed and cleaved. However, our data suggest that the differences in nucleophile reactivity and structure can be readily accommodated by TEV protease with apparently little trade-off between rates for different nucleophiles. The unexpected nucleophile tolerance suggests that chemically versatile intermediates such as TEV^Ser/Cys^X exist that could facilitate the phylogenetically observed switch between protease clans that differ in their nucleophiles prior to specialisation. The protein framework of TEV^Ser/Cys^X allows two nucleophiles to execute their function with good efficiency and constitutes a molecular solution to escape from adaptive conflict.

## Supporting Information

As a service to our authors and readers, this journal provides supporting information supplied by the authors. Such materials are peer reviewed and may be re-organized for online delivery, but are not copy-edited or typeset. Technical support issues arising from supporting information (other than missing files) should be addressed to the authors.

miscellaneous_information
