# Supplementary material for: Handicap-Recover Evolution Leads to a Chemically Versatile, Nucleophile-Permissive Protease
Source: Chembiochem. 2015 Jul 14;16(13):1866–9. doi: 10.1002/cbic.201500295 (PMC4576821; doi:10.1002/cbic.201500295)
Supplement: Supplementary file 1 — miscellaneous_information [file cbic0016-1866-sd1.pdf]

CHEMBIOCHEM

Supporting Information

Handicap-Recover Evolution Leads to a Chemically Versatile, Nucleophile-Permissive Protease

Thomas Shafee,[a, b, c] Pietro Gatti-Lafranconi,[a] Ralph Minter,[b] and Florian Hollfelder*[a]

cbic_201500295_sm_miscellaneous_information.pdf

Contents

Methods 2
Phylogeny 2
Directed mutagenesis 2
Random mutagenesis 2
96-well plate screening 2
Protein Expression and Purification 2
In vitro kinetics 3
List of Plasmids used in this work 4
List of Primers used in this work 4

Kinetic analysis 5

Supplementary Tables 7
Supplementary Table 1 7
Supplementary Table 2 8
Supplementary Table 3 9

Supplementary Figures 10
Supplementary Figure 1 10
Supplementary Figure 2 11
Supplementary Figure 3 12
Supplementary Figure 4 13
Supplementary Figure 5 14
Supplementary Figure 6 16

Supplementary References 17

Methods

Phylogeny

An alignment based on structural homology to TEV protease (PDB 1lvm) was generated using DALI protein.[1] From this primary homology assessment, sequences lacking known structures were added based on amino acid sequence similarity to several proteases from the phylogeny using BLASTp (see Supplementary table 1 for details and additional methods). The amino acid sequences were aligned with T-Coffee[2] and used to generate a maximum likelihood phylogeny in MEGA5.[3]

Directed mutagenesis

Primer T_M was calculated using NetPrimer (standard conditions, 1.5 nM Mg^{2+}). Two PCR reaction using flanking primers and primers containing the desired C151S mutation (primers tevF+serR and tevR+serF, respectively) were performed (10 rounds of: 30 s at 94 °C, 30 s at (T_M-5) °C, 60 s at 72°C) using 0.4 u Pfu Turbo (Agilent), each primer (1 µM), dNTPs (2.5 mM), template plasmid (100 ng in 20 µL). The two amplified products were mixed and overlap extension PCR with the flanking primers tevF+tevR completed the gene (10 rounds of: 30 s at 94 °C, 30 s at (T_M-5) °C, 60 s at 72°C) using 0.4 u Pfu Turbo (Agilent), each primer (1 µM), dNTPs (4 mM) and linear DNA template (100 ng in 20 µL). The product was ligated into double-digested plasmid pMAA and then used to transform electro-competent *E. cloni* (lucigen) as per manufacturer's instructions. See Supplementary File 1 for the full list of primers and plasmids used in this work.

Random mutagenesis

Error prone PCR using flanking primers tevF+tevR was performed (30 rounds of: 30 s at 94 °C, 30 s at 50 °C, 90 s at 72 °C, 30 rounds) using 0.4 u Mutazyme II polymerase (Agilent), each primer (1 µM), dNTPs (0.8 mM), template plasmid (400 ng in 20 µL.) The product was ligated into pMAA as described above.

96-well plate screening

E. coli cells (TOP10; Invitrogen) were transformed according to the manufacturer's instructions with pC-Y (containing gene for C-Y FRET substrate) and pMAATEVlib (containing the library of TEV variants produced by mutagenesis).

In a 96-deepwell plate, LB (1 mL per well, containing the appropriate antibiotics) was inoculated with the transformed cells to be tested and incubated for 12 hours at 30 ˚C. The cells were induced by addition of 50 µL L-arabinose (0.2% final concentration) and incubated at 25 °C for a further 6 hours. Cells were pelleted, then resuspended in PBS + BugBuster® (250 µL; Novagen) and incubated for 1 hour at 25 °C. Finally, cell debris were pelleted and the fluorescence of the supernatant assayed in a Spectramax M5 (Molecular Devices, excitation 414 nm, emission 475 nm and 525 nm). The FRET efficiency ratio was calculated as the emission at 525 nm divided by the emission at 475 nm.

Protein Expression and Purification

For both C-Y and TEV variants, LB (1 L, containing the appropriate antibiotics) was inoculated with cells (from an overnight inoculum) and incubated at 37 °C and 200 rpm for 4 hours. The cells were induced by addition of L-arabinose (0.2%) and incubated at 30 °C for a further 4 hours. The cells were pelleted and frozen for storage. The cell pellet was thawed and resuspended in 10 mL lysis buffer (50 mM Tris, 300 mM NaCl, 5 mM imidazole, pH 8). Cells were lysed on ice by sonication (2s on, 10s off, 5min) and pelleted. The supernatant was loaded onto a HisTrap (GE Life Sciences, 5ml) column, washed with the same buffer except that imidazole concentration

was 50 mM and then eluted with 300mM imidazole in 500µL fractions. Protein content was monitored by A_{280} and checked by gel electrophoresis. Fractions containing the protein of interest were pooled and exchanged into TEV Storage buffer (50 mM Tris, 1 mM EDTA, 10% sucrose, pH 8) using a HiTrap desalting column (GE Life Sciences, 5 mL). 100 µL aliquots were snap-frozen using liquid nitrogen.

In vitro kinetics

Purified cyan- and yellow-fluorescent protein fusion substrate (linked by the canonical sequence ENLYFQS, see Supplementary Figure 4) was thawed overnight at 4 ˚C. Unless otherwise stated, 1 µM substrate was added to enzyme (1- 8 µM) in 200 µL TEV buffer (50 mM Tris, 1 mM EDTA, 10% sucrose, pH 8) including freshly made-up DTT (1 mM) in a 96-well plate. Solutions were assayed in a Spectramax M5 (Molecular Devices, excitation 414 nm, emission 475 nm and 525 nm) for 12 hours at 25 ˚C. Progress curves were fit to a single exponential model used previously[4] or, in the case of biphasic kinetics, fit to a modified two-exponential model (Supplementary Figure 3).

List of Plasmids used in this work

NAME	DETAILS
**pMAA-MBPTEV**	Arabinose-inducible expression of MBP-Tc-TEV fusion (Tc = Thrombin cleavage site)
**pC-Y**	Arabinose-inducible plasmid encoding a CFP and YFP fusion[5] separated by a linker with sequence GGSGS**ENLYFQS**GSGGS (TEV protease cleavage site in bold)

List of Primers used in this work

NAME	SEQUENCE (mutagenic nucleotides in lower case)
**tevF**	GTTCCGAGGGGATCCATGGG
**tevR**	CCCCGATCCCTCGAGAAGC
**serF**	GGGCAGTcTGGatccCCATTAGTATCAACTAGAGATGGG
**serR**	CTAATGGggatCCAgACTGCCCATCCTTGGTTTGAATCC

Kinetic analysis

For consistency between the *in vivo* assay used for directed evolution and *in vitro* studies with purified components, all kinetic analysis was performed using the CFP-ENLYFQS-YFP construct which contains the native TEV cleavage sequence. This substrate is referred to as C-Y throughout the manuscript.[5]

The use of the same substrate for TEV protease *in vitro* for kinetics after purification (Supplementary Figure 3 and Supplementary Figure 4) as well as *in vivo* for directed evolution screening (Supplementary Figure 2) made it possible to correlate the measured rates with the behavior under selection conditions (directly reflecting the evolutionary pressure applied).

Kinetics were consistently measured under conditions of sub-saturating substrate (with [E] in a similar range as [S]: [S] = 1 µM and [E] = 4 µM (TEV^{Cys}) or 20 µM (TEV^{Ser})), so that they represent k_{cat}/K_M differences (see further evidence below). Rate constants were obtained by fitting time courses of product formation (to equations shown in Supplementary Figure 3), similar to an approach used previously for quantifying protease activity.[4] TEV^{Ser} and variants evolved from TEV^{Ser} failed to fit to the single exponential kinetic model used by Boulware and Daugherty,[4] but could be fitted well to a model involving two exponential reactions (Supplementary Table 3), consistent with a fast first step followed by a rate limiting second step. The pseudo-first order rate constants obtained (with eq. 1 or 2, as reported above) were divided by the enzyme concentration to give a pseudo-second order rate constant (at the specific substrate concentration of 1 µM). Evidence for these second order rate constants to reflect relative k_{cat}/K_M values is the linearity of the fitted rates with substrate concentration under the experimental conditions. Likewise, the pseudo-first order rate constants were also proportional to enzyme concentration.

The *second order* rate constants obtained were used for comparison of TEV^{Cys} and TEV^{Ser} variants. It should be noted that no argument is based on an interpretation of the observed burst kinetics to correspond to specific elemental steps, as all plausible scenarios involve the nucleophile that is changed in this work. Thus the validity of any tentative explanations in this section do not affect any of the conclusions in the main text.

There are multiple possibilities to assign the apparent steps observed in time courses. The monophasic kinetics of TEV^{Cys} suggest one rate-limiting, irreversible step, e.g. the formation of an acyl-enzyme intermediate. Later steps that may be present would have to be much faster, making them kinetically invisible under these conditions. In some mutants of TEV^{Ser} (Supplementary Table 3 and Supplementary Figure 3) the time courses fit poorly to the single exponential equation, but fit well to a two exponential model. This is consistent with a later step becoming slow enough to be visible and limiting the rate of the overall reaction. The burst amplitude increases linearly with enzyme concentration (Supplementary Figure 5), consistent with the amplitude representing the formation of a covalent intermediate. In our data the burst amplitude varies (Supplementary Table 3) between 0.01 and 0.1 x [E]. This situation is similar to many reported examples where the burst amplitude does not equal [E], either below or above [E].[6,7] Burst phases that do not equal the enzyme concentration can be the result of internal equilibria and competition between rates of product formation and product release.[8–10] For example, fast product release leads to a faster approach to steady-state and decreases the burst amplitude. If the burst generates product concentrations above K_P, the linear phase underestimates real rates and leads to larger burst amplitudes. We note that as k^{obs2} increases in a multistep reaction, the burst amplitude decreases.

The trends observed *in vivo* and *in vitro* give an identical rank order of TEV mutant activity, although the magnitude of the observed changes differs (10-fold *in vivo vs* 10^4-fold *in vitro*). Therefore the *in vivo* assay is good predictor for 'activity' and useful for selections. However, it does not mean that all positive variants would have the same *in vitro* activity, which likely reflects an endpoint rather than kinetic readout, leading to the difference in dynamic ranges.

Using the a C-Y protein substrate for kinetics under Michaelis-Menten conditions ([E]<<[S]) was not possible, due to its limited solubility at high concentrations, where saturation might be expected. Therefore only relative second order rate constants can be reported. The substrate used for the ratiometric FRET assay allowed meas-

urements in a limited dynamic range, so the need to use sufficient enzyme concentrations to detect visible turnover disallowed lowering [E] to an extent that [E]<<[S] conditions could be restored. Soluble small-molecule substrates were ruled out as experimental alternatives as they do not reflect the C-Y substrate used for evolution experiments and exhibit different kinetic behavior to protein substrates.[4] Furthermore, since the proteases studied in this work follow a multistep reaction, Michaelis-Menten parameters k_{cat} and K_M do not necessarily report on straightforwardly interpretable elemental chemical or binding steps, so no further systematic attempt was made to determine k_{cat} and K_M separately.

Supplementary Tables

Supplementary Table 1

Sequences added to the phylogeny by a combination of structure- and sequence-based database searches

Blast query	*PDB*	*No. sequences added*
TEV	1lvm	75
Astrovirus	2w5e	14
Rhinovirus 2A*	2hrvB	14
Sesbania mosaic	1zyo	12
Rhinovirus 3C*	1cqqA	10
Alkaline protease	3cp7	8
Norovirus	2fyq	6
Achromobacter	1arc	5
Hepititus C	2obq	4
Human BB fragment	1rtk	3
Equine arteritis	1mbm	3
Hepatitis A	1hav	2

Supplementary Table 1. Alignment based on structural similarity (DALI server)[1] gave better alignment of diagnostic active site residues (e.g. the catalytic triad), secondary structure elements, and gap placement for the primary homology assessment. Initially, a DALI alignment was performed using TEV as reference. The top 12 structures isolated on the basis of structural similarity are listed in this table. As these share <30% sequence identity with TEV, the number of sequences used for the phylogeny was expanded by running BLASTp searches with each of the 12 protein sequences. The table indicates the number of protein sequences with unknown structure (but identity to the corresponding search query >30%) that have been added to the phylogeny according to this procedure. Sequences cluster in groups that correspond to known subfamilies within the PA clan (e.g. S1 and C3 families). *Rhinovirus has two paralogous copies of its protease. Protease 2A is smaller than 3C and has a different substrate specificity

Supplementary Table 2

Round	Mutations	k_2^{obs1} / (min^{-1}M^{-1}) [a]	k_2^{obs2} / (min^{-1}M^{-1}) [a]	Solubility [b]	k_2 (min^{-1}M^{-1}) [a]	Solubility [b]
		Serine nucleophile (TEV^{Ser})			Cysteine nucleophile (TEV^{Cys})	
TEV		$3 \bullet 10^2$	9×10^{-1}	89%	$2 \bullet 10^4$	100%
I	**K6N, R50G, E223G**	$2 \bullet 10^4$	$5 \bullet 10^1$	70%	$1 \bullet 10^4$	66%
II	K6N, R50G, **T113S**, E223G, **A231V**	$7 \bullet 10^4$	$3 \bullet 10^2$	40%	$2 \bullet 10^4$	38%
III	K6N, R50G, T113S, **K215R**, E223G, A231V	$5 \bullet 10^4$	$3 \bullet 10^2$	46%	$1 \bullet 10^4$	25%
IV	K6N, **H28L**, R50G, T113S, **C130W**, **L210M**, K215R, E223G, A231V	$2 \bullet 10^5$	$2 \bullet 10^2$	18%	$1 \bullet 10^4$	11%
V	K6N, H28L, R50G, T113S, **W130C,** L210M, K215R, E223G, A231V	$4 \bullet 10^5$	$1 \bullet 10^3$	39%	$2 \bullet 10^4$	25%
VI	K6N, H28L, R50G, T113S, **E194D**, L210M, K215R, E223G, **(Frameshift*)**	$3 \bullet 10^5$	$2 \bullet 10^3$	44%	$6 \bullet 10^4$	30%
VII	K6N, H28L, R50G, T113S, E194D, **A206T**, L210M, K215R, E223G, (Frameshift*)	$4 \bullet 10^5$	$2 \bullet 10^3$	29%	$8 \bullet 10^4$	30%
VIII	K6N, H28L, R50G, T113S, **N174K**, E194D, A206T, L210M, K215R, E223G, (Frameshift*)	$4 \bullet 10^5$	$7 \bullet 10^2$	30%	$2 \bullet 10^4$	19%
IX	K6N, H28L, **I35L**, R50G, T113S, **L155V**, N174K, E194D, A206T, L210M, K215R, E223G, (Frameshift*)	$6 \bullet 10^5$	$1 \bullet 10^2$	23%	$3 \bullet 10^4$	24%
X	K6N, H28L, **S31T**, I35L, R50G, T113S, L155V, N174K, E194D, A206T, L210M, K215R, E223G, (Frameshift*)	$7 \bullet 10^5$	$3 \bullet 10^3$	15%	$7 \bullet 10^3$	14%

[a] Measured as k^{obs1}/[E]. Interpreted previously as k_{cat}/K_M of TEV.[4] [b] Soluble expression was measured in % of wt expression.

Supplementary Table 2. Characterization of TEV mutants obtained in evolution experiments. Mutations accumulated in each round (new mutations in bold). Kinetics were measured with purified TEV enzyme and C-Y fusion substrate under sub-saturating conditions (cleavage of 1 µM substrate by 15-20 µM TEV^{Ser} and 1-8 µM of all other enzyme variants). Rate constants were obtained by fitting time courses to single (Cys variants) or double (Ser variants) exponential equations to give one (k^{obs1}) or more (k^{obs2}) first order rate constants. These first order rate constants were divided by the enzyme concentration to give second order rate constants (k_2^{obs1} or k_2^{obs2}) that reflect changes in second order rates k_{cat}/K_M (see Supplementary Figure 3)[4] *Mutations K229R, E230K and Δ231-236 are all caused by a one nucleotide frameshift. The FRET efficiency ratio was calculated as the emission at 525 nm divided by the emission at 475 nm after excitation at 414 nm). *Conditions: pH 8, T = 25 °C.*

Supplementary Table 3

Serine Variants	*R^2 of fit*		*Cysteine Revertants*	*R^2 of fit*	
	Eqn. 1	Eqn. 2		Eqn. 1	Eqn. 2
TEV^{Ser}	0.92	0.98	TEV^{Cys}	0.99	0.99
TEV^{Ser}I	0.92	0.96	TEV^{Cys}I	0.98	0.99
TEV^{Ser}II	0.91	0.98	TEV^{Cys}II	0.98	0.98
TEV^{Ser}III	0.86	0.98	TEV^{Cys}III	0.99	0.99
TEV^{Ser}IV	0.90	0.96	TEV^{Cys}IV	0.99	0.99
TEV^{Ser}V	0.73	0.98	TEV^{Cys}V	0.99	0.99
TEV^{Ser}VI	0.31	0.97	TEV^{Cys}VI	0.99	0.99
TEV^{Ser}VII	0.26	0.98	TEV^{Cys}VII	0.99	0.99
TEV^{Ser}VIII	0.83	0.95	TEV^{Cys}VIII	0.95	0.98
TEV^{Ser}IX	0.81	0.94	TEV^{Cys}IX	0.95	0.99
TEV^{Ser}X	0.54	0.97	TEV^{Cys}X	0.93	0.99

Supplementary Table 3. Assessment of the goodness of fit for fits to single or double exponential model (equation 1 or equation 2, respectively) for all DE variants and their S151C nucleophile revertants. Curve fitting was performed using Excel Solver iteration plug-in (see Supplementary Figure 3). Cleavage of 1 µM substrate by 15-20 µM TEV^{Ser} and 1-8 µM of all other enzyme variants, pH 8, T = 25 °C, excitation = 414 nm, FRET ratio was calculated as emission 525 nm / 475 nm as in reference.[5] The pseudo-first order rate constants obtained by eq. 1 or 2 were divided by the enzyme concentration to give a pseudo-second order rate constant (at the specific substrate concentration of 1 µM). The goodness-of-fit for either model (eq. 1 or eq. 2) is shown in the Table. The fit with the higher value of R^2 was used to determine the data in Table 1. Broadly, equation 1 fits the time courses of later TEV^{Cys} revertants less well, suggesting that in the first reaction step may be becoming increasingly rate limiting.

Supplementary Figures

Supplementary Figure 1

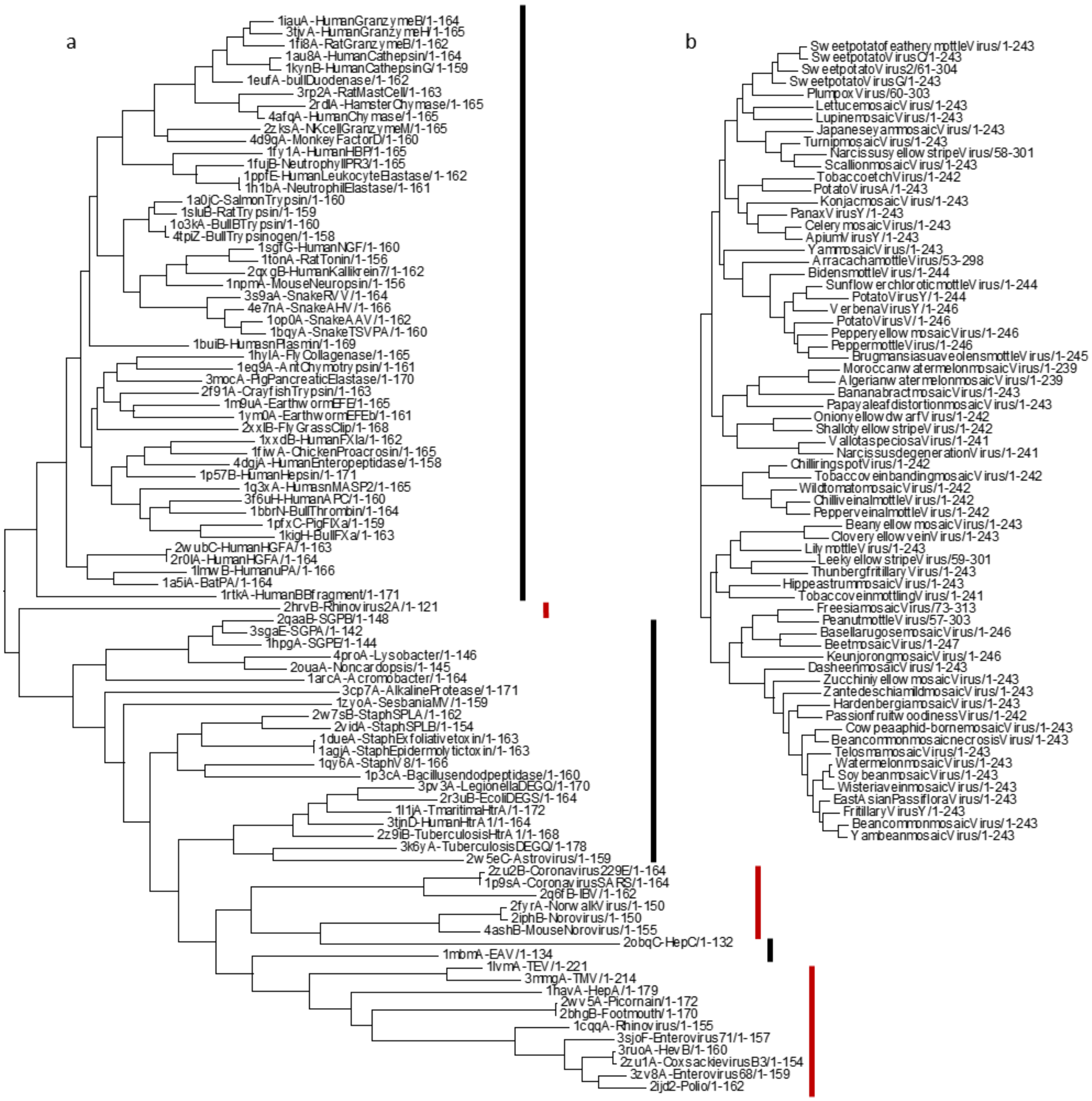

Supplementary Figure 1. Phylogenies based on structural alignment or sequence alignment only. Maximum likelihood phylogenies of sequences (a) of all structurally characterized PA clan proteases identified and aligned by DALI from TEV protease structure query and (b) of viral proteases in the C3 family with sufficient sequence similarity to be identified by BLASTp and aligned by T-Coffee from TEV protease sequence query. Red lines indicate cysteine nucleophiles, black indicates serine nucleophiles. The alignments used to generate these phylogenies were combined, along with similar sequence alignments detailed in Supplementary Table 1 to generate the alignment on which Figure 1b is based.

Supplementary Figure 2

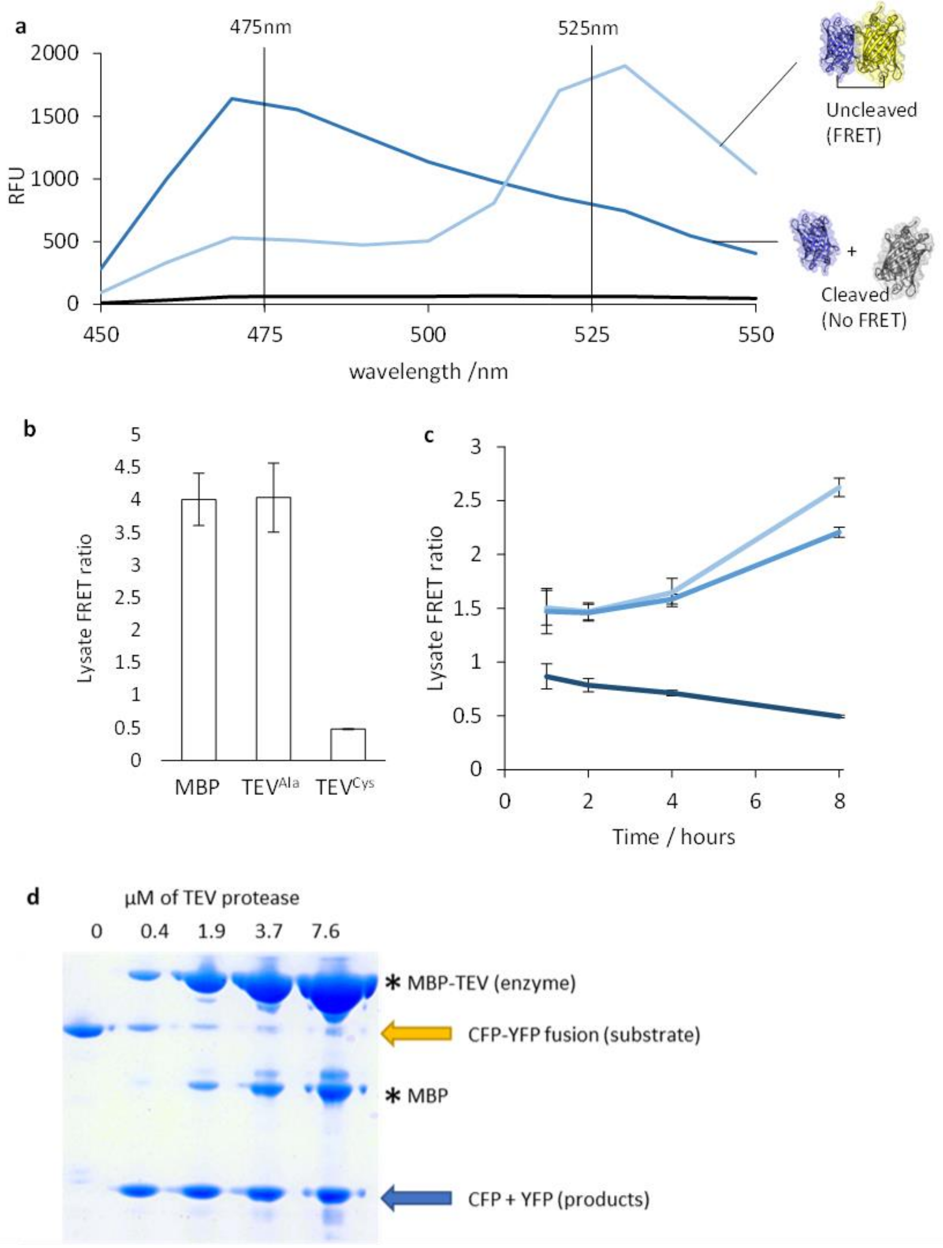

Supplementary Figure 2. Hydrolysis of the C-Y FRET pair substrate by TEV^{Cys}. (a) Emission spectra of cleared lysates after 4hour co-expression of substrate and enzyme (excitation = 414 nm). Expression of C-Y FRET substrate and MBP (light blue), C-Y and TEV^{Cys} (dark blue), TEV^{Cys} only (black). Cleared lysates were assayed in a Spectramax M5 (Molecular Devices). (b) Cleared lysate FRET ratios after 4 hour co-expression of C-Y FRET substrate with maltose binding protein (MBP), TEV C151A (TEV^{Ala}) or TEV^{Cys}. The FRET ratio was calculated as the fluorescence value measured at 525 nm/475 nm. A *lower* ratio indicates higher proteolytic activity.[5] The lack of activity by TEV^{Ala} indicates that binding does not affect FRET (c) FRET ratios of cleared lysates after 1, 2, 4 & 8 hour co-expressions. Expression of C-Y and MBP (light blue), C-Y and TEV^{Cys} (dark blue), C-Y and TEV^{Ser} (blue). (d) Cleavage of 1.1 μM of C-Y substrate by different amounts of TEV^{Cys} protease. Purified C-Y substrate was thawed overnight at 4 ˚C and the reaction was incubated for 4 h at 21°C. Asterisks indicate the position of MBP-TEV fusion and of the minor autoproteolysis product, MBP. Arrows indicate the position of the GFP fusion pair (yellow) and of the cleaved individual GFP variants (blue).

Supplementary Figure 3

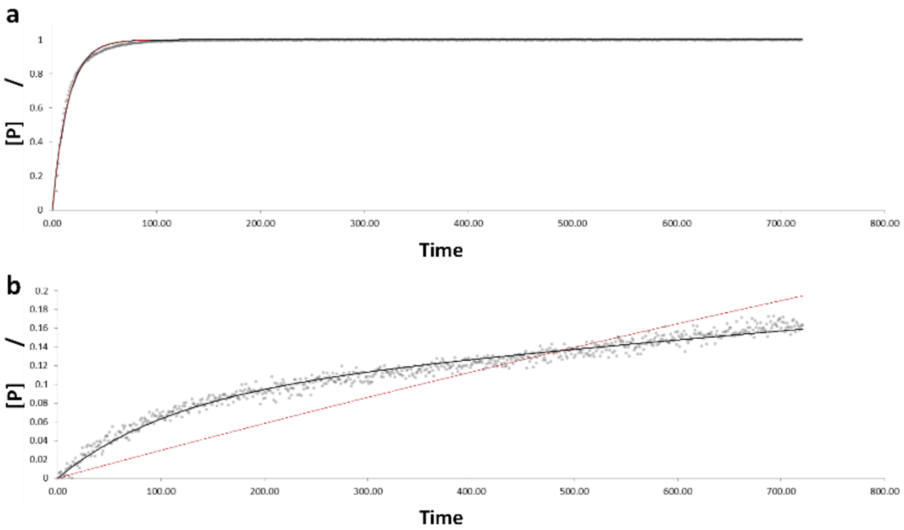

Supplementary Figure 3. Kinetic measurements were interpreted as outlined by Boulware and Daugherty.[4] The wild-type kinetics were monophasic and could fit to the single exponential equation 1 and a representative time course is shown Eq 1, is limited to kinetics in which the first step is rate-limiting (as for TEV^{Cys} k_{cat}/K_M). Therefore the modified Eq 2 was used for TEV^{Ser}, as it allows fitting of rates both before (k^{obs1}) and after (k^{obs2}) the rate-limiting step (R^2 fits to Eq 1 and Eq 2 for TEV^{Cys} and TEV^{Ser} are compared in Supplementary Table 2).

Examples of fitting mono- and biphasic models to kinetic traces for cleavage of the substrate C-Y by TEV protease variants. (a) Wild-type TEV^{Cys} (4 µM), (b) TEV^{Ser} (20 µM), [substrate] = 1 µM, pH 8, T = 25 °C. Purified C-Y substrate was thawed overnight at 4 ˚C. The FRET ratio was calculated as ratio of emission 525 nm and 475 nm after excitation at 414 nm. Kinetics were consistently measured under conditions of sub-saturating substrate (with [E] in a similar range as [S]: [S] = 1 µM and [E] = 1 - 8 µM), so that they represent k_{cat}/K_M differences as in reference.[4] The wild-type kinetics were monophasic and could fit to the single exponential equation 1 and a representative time course is shown in panel a. TEV^{Ser} and mutants evolved from it did not give reasonable fits to a single exponential equation (eq. 1). A representative time course is shown in panel b. In addition to the (obviously unsuitable) fit to a single exponential in red (eq. 1), a double exponential fit according to equation 2 is shown in black:

Equation 1. $[P] = [P]_{max} - e^{-[E]\frac{k_{cat}}{K_M}t}$

Where [P] = concentration of product, $[P]_{max}$ = final concentration of product (approximates starting substrate concentration), A_1 = first burst amplitude (interpreted as the fractional turnover performed in first catalytic step), [E] = concentration of enzyme, k_{cat}/K_M = rate constant of the reaction and t = time.

Equation 2. $[P] = [P]_{max} - \left(A_1 e^{-[E]k_{obs1}t} + A_2 e^{-[E]k_{obs2}t}\right)$

Where [P] = concentration of product, $[P]_{max}$ = final concentration of product (approximates starting substrate concentration), A_1 = first burst amplitude (interpreted as the fractional turnover performed in first catalytic step), A_2 = Second burst amplitude (should equal $[P]_{max}$-A1), [E] = concentration of enzyme, k^{obs1} = rate of first catalytic step, k^{obs2} = rate of second catalytic step and overall reaction and t = time.

Supplementary Figure 4

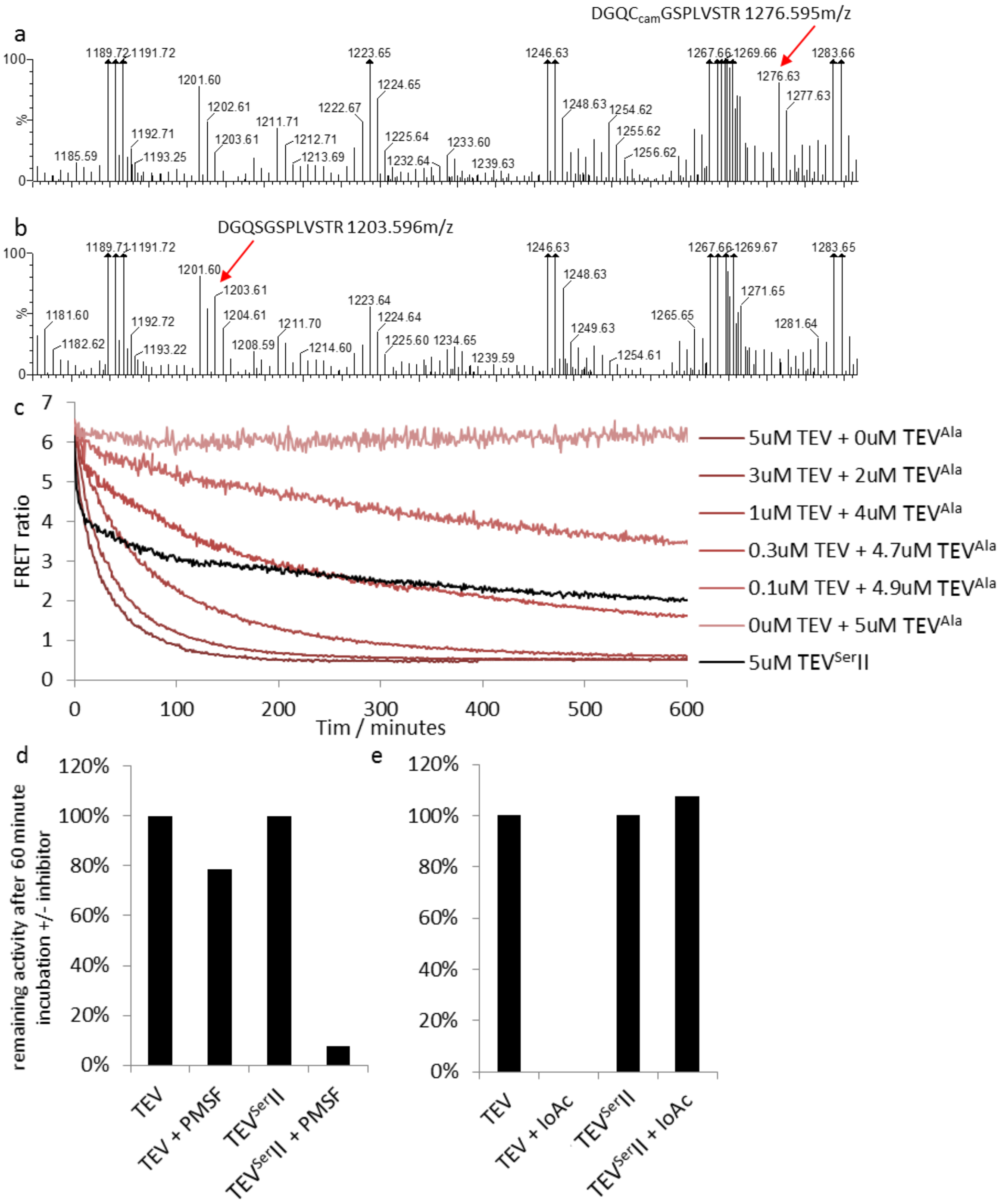

Supplementary Figure 4. Characterization of the catalytic serine of TEV^{Ser} variants. Mass spectrometry confirmed (a) the presence of a Cys-containing fragment in TEV and (b) the presence of a Ser-containing fragment in TEV^{Ser}. Kinetic profiles on 1 µM C-Y substrate (c) show that mixtures of mostly inactive enzyme (TEV^{Ala}), with a minority of active enzyme (TEV) fails to recreate the biphasic kinetics of the TEV^{Ser} variants. Inhibition of 5 µM TEV and TEV^{Ser}II by (d) 10 µM phenylmethane sulfonyl fluoride (a serine protease inhibitor) and (e) 10 µM iodoacetate (a cysteine protease inhibitor) confirms that activity is dependent on the active site serine in the TEV^{Ser} variants.

Supplementary Figure 5

A

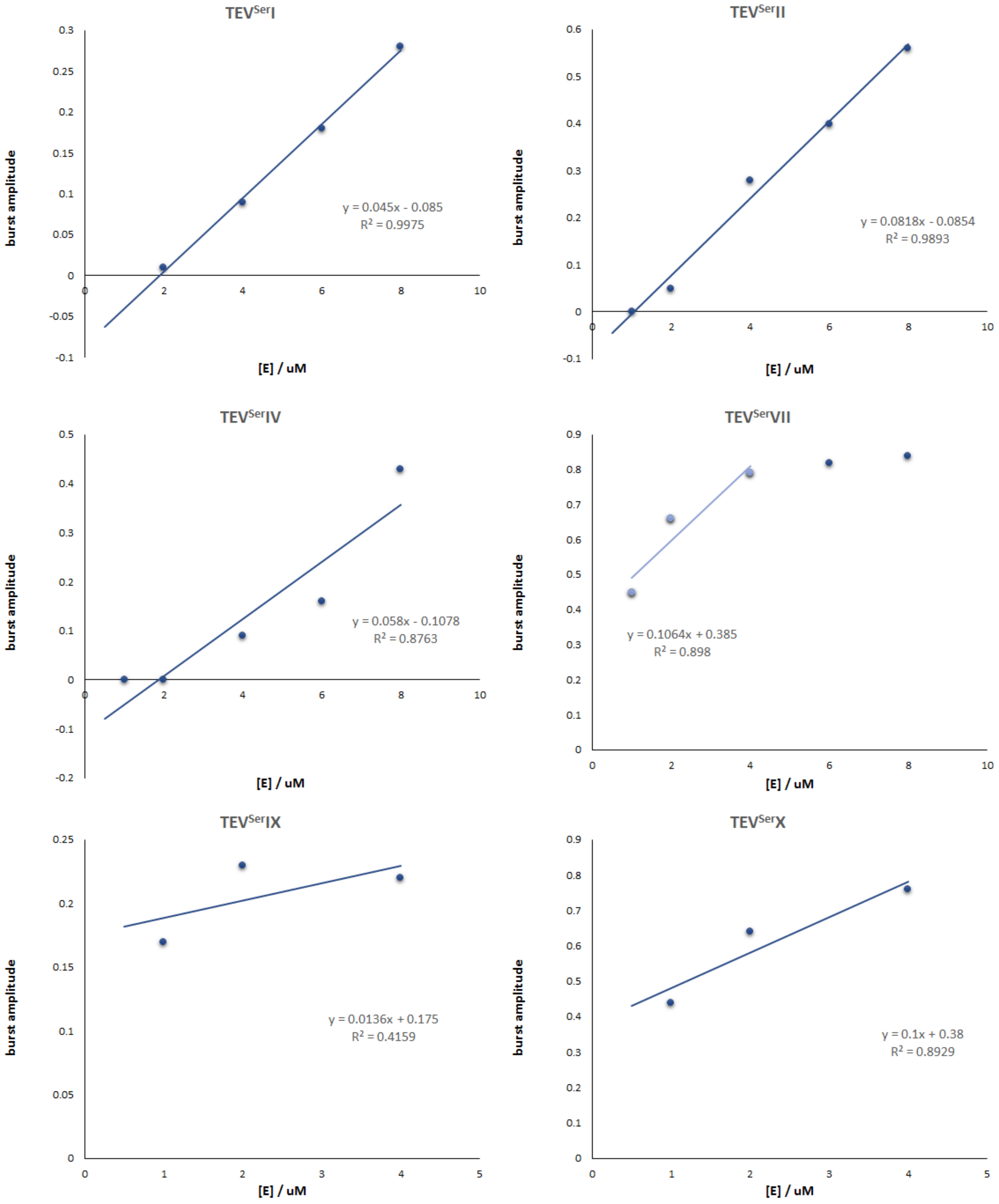

B

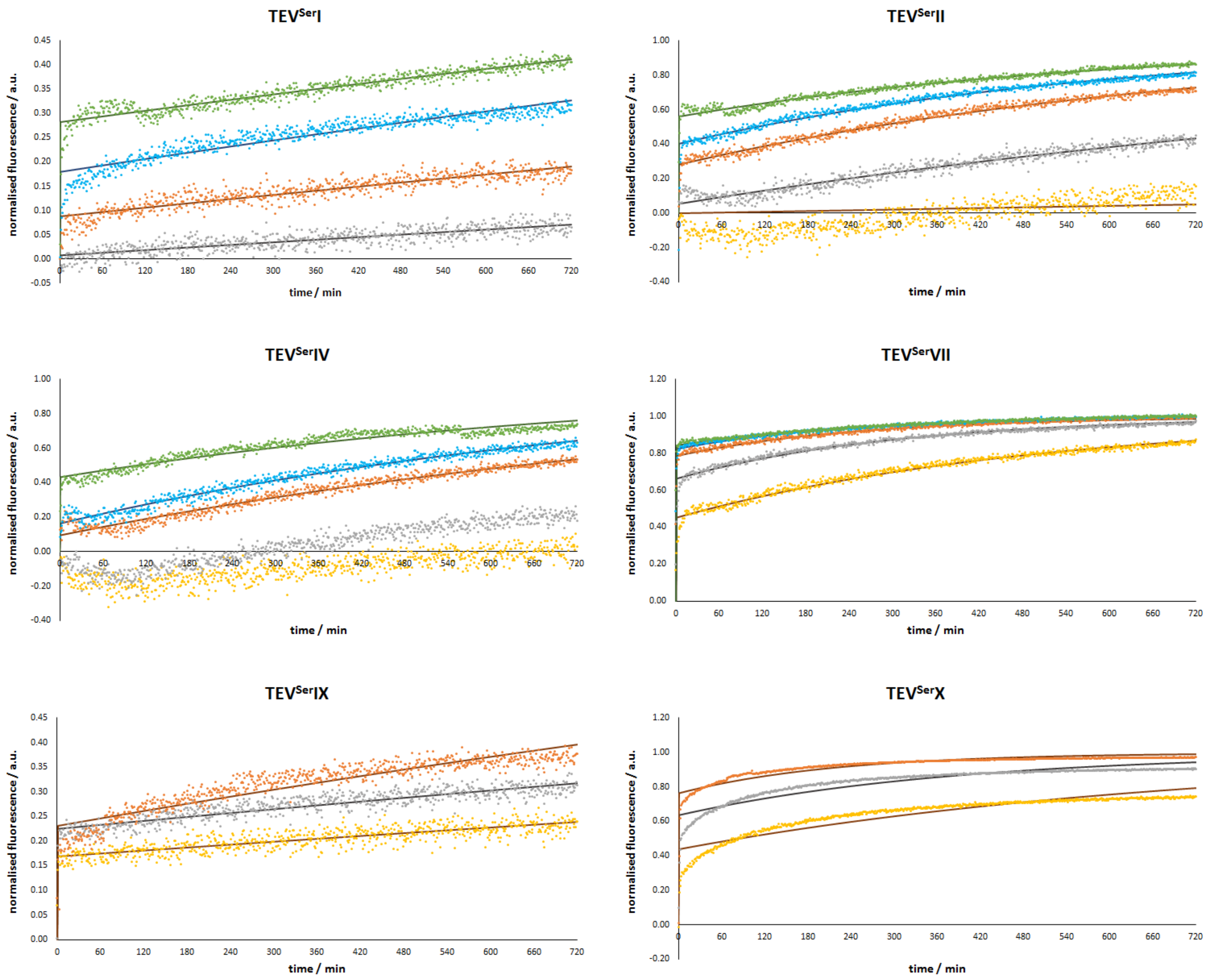

Supplementary Figure 5. (A) The observation of a burst is consistent with the existence of an intermediate. (A) Plots of the burst amplitude (as measured using eq.2, Supplementary Figure 3) against the enzyme concentration are linear. (B) The model generated by eq. 2 (solid lines) is overlaid on the corresponding experimental data. These curves have been used to extract the burst amplitude values plotted in panel A. The range of enzyme concentrations used was: 1 mM (yellow), 2 mM (grey), 4 mM (orange), 6 mM (cyan) and 8 mM (green). The concentration of C-Y substrate was kept constant at 1 mM. For TEV^{Ser}VII the deviation from linearity was observed in the non-linear region of the ratiometric assay and thus disregarded.

Supplementary Figure 6

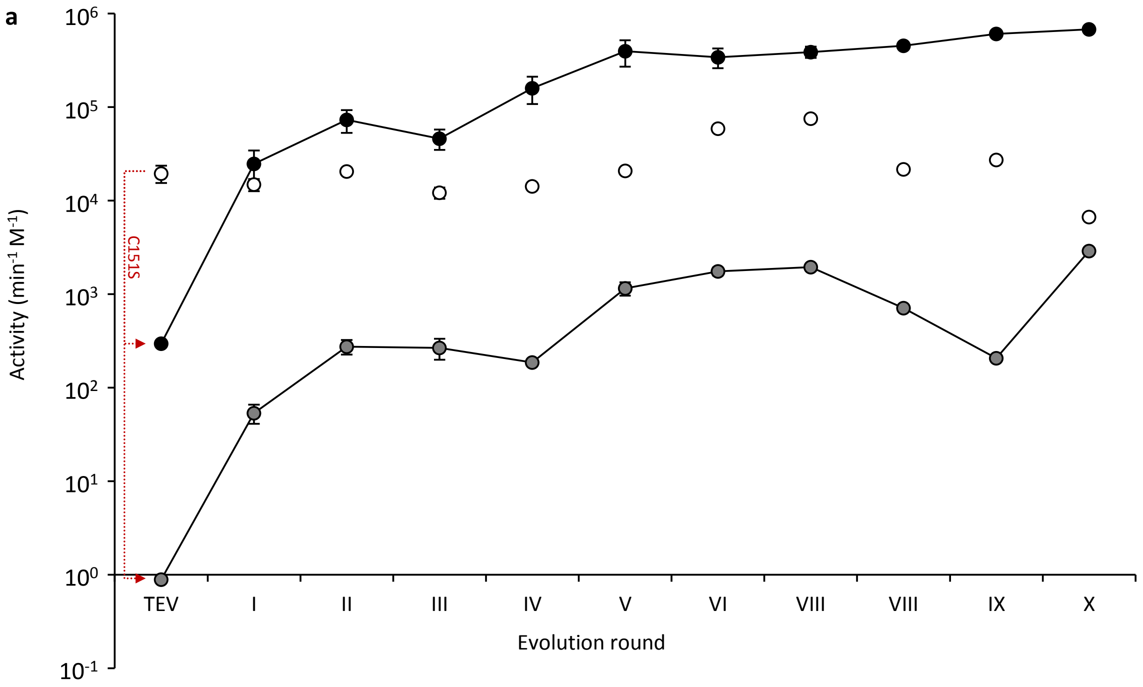

Supplementary Figure 6. *Nucleophile activity trade-off*. (a) Kinetic activity of purified enzyme with 1 µM substrate (pH 8, 25°C). Shown are k_{cat}/K_M for TEV^{Cys} variants (white circles) and k_2^{obs1} and k_2^{obs2} for TEV^{Ser} variants (black and grey circles respectively). The red dashed arrow indicates the effect of the initially introduced handicap nucleophile mutation (C151S). Error bars indicate standard deviation of 3-4 repeats at enzyme concentrations (15-20 µM TEV^{Ser} and 1-8 µM of all other enzyme variants).

Supplementary References

1. Holm, L. & Rosenström, P. Dali server: conservation mapping in 3D. *Nucleic Acids Res.* **38,** W545–9 (2010).

2. Notredame, C., Higgins, D. G. & Heringa, J. T-coffee: a novel method for fast and accurate multiple sequence alignment. *J. Mol. Biol.* **302,** 205–217 (2000).

3. Tamura, K. *et al.* MEGA5: molecular evolutionary genetics analysis using maximum likelihood, evolutionary distance, and maximum parsimony methods. *Mol. Biol. Evol.* **28,** 2731–9 (2011).

4. Boulware, K. T. & Daugherty, P. S. Protease Specificity Determination by Using Cellular Libraries of Peptide Substrates (CLiPS). *Proc. Natl. Acad. Sci.* **103,** 7583–7588 (2006).

5. Nguyen, A. W. & Daugherty, P. S. Evolutionary optimization of fluorescent proteins for intracellular FRET. *Nat. Biotechnol.* **23,** 355–60 (2005).

6. Escobar, W. A., Tan, A. K., Lewis, E. R. & Fink, A. L. Site-Directed Mutagenesis of Glutamate-166 in .beta.-Lactamase Leads to a Branched Path Mechanism. *Biochemistry* **33,** 7619–7626 (1994).

7. Jonas, S., van Loo, B., Hyvönen, M. & Hollfelder, F. A new member of the alkaline phosphatase superfamily with a formylglycine nucleophile: structural and kinetic characterisation of a phosphonate monoester hydrolase/phosphodiesterase from Rhizobium leguminosarum. *J. Mol. Biol.* **384,** 120–36 (2008).

8. Purich, D. L. *Enzyme Kinetics and Mechanism: Part F: Detection and Characterization of Enzyme Reactions*. (2002).

9. Copeland, R. A. *Enzymes: A Practical Introduction to Structure, Mechanism, and Data Analysis*. *Enzymes: A Practical Introduction to Structure, Mechanism, and Data Analysis* (2004).

10. Raton, B. *Mechanism-Based Enzyme Inactivation: Chemistry and Enzymology*. (1988).
